# Glycation and oxidative stress in the failure of dental implants: a case series

**DOI:** 10.1186/1756-0500-6-296

**Published:** 2013-07-26

**Authors:** Davide Pietropaoli, Eleonora Ortu, Marco Severino, Irma Ciarrocchi, Roberto Gatto, Annalisa Monaco

**Affiliations:** 1 University of L’Aquila - Department of Life, Health and Environmental Sciences San Salvatore Hospital, Building Delta 6 – Unit of Dentistry - Via Vetoio, L’Aquila, 67100, Italy

**Keywords:** Dental implants, Periimplantitis, Oxidative stress, Glycation, Advanced glycation end products, AGEs, ROS

## Abstract

**Background:**

The aim of this case series/control study is to investigate the presence of the Advanced Glycation End products (AGEs) and oxidative stress in periimplantitis.

The study group was composed of five dental implants, failed within 6 months after implantation, taken from 5 subjects (3 M/2 F) aged between 43–57 years and stored in isotonic liquid before freezing at -80°C, according to literature. All the implants had been placed using traditional submerged technique. The whole saliva was also collected using Salimetrics device and stored at -80°C, to assess molecular analysis. Two age-matched control groups were examined: they consisted of 5 subjects encountering dental extraction for chronic periodontal disease (2 M/3 F) and 5 healthy subjects (3 M/2 F) who needed extraction for dental trauma. Their whole saliva was collected with the same method. The implants and the tooth of control groups were processed to assess Western Blotting for identification of AGEs. The case/control whole saliva was used to perform ThioBarbituric Acid Reactive Substances (TBARS) for oxidative stress evaluation.

**Findings:**

The Western Blotting analysis on periimplantitis and periodontal disease tissues showed marked increase of AGEs when compared to healthy control tissues. Also TBARS assay of whole saliva confirmed the expectations, showing higher oxidative stress levels in periimplantitis and periodontitis groups than in healthy group.

**Conclusions:**

With the limitation of the sample size, these results showed that oxidative stress could be involved in the aetiology of periimplantitis. This hypothesis could lead to new therapeutic strategies in periimplantitis, using antioxidant approach in addition to conventional treatments.

## Findings

To date it is commonly accepted that the failure of dental implants can be defined as the inability of tissue to establish or maintain osteointegration, caused by host response and opportunistic infection. In fact, the Sixth European Workshop on Periodontology in 2008 has confirmed that “*peri-implant diseases are infectious in nature. Peri-implant mucositis describes an inflammatory lesion that resides in the mucosa, while periimplantitis also affects the supporting bone*” [1].

To date many studies suppose that oxidative stress plays and important role in the aetiology and severity of periodontal diseases, but there are no researches in this field for periimplantitis. We suppose that the same mechanisms are involved in the failure of dental implants.

Substantially, the periodontal bacteria promote the flogistic events that lead to an increase in intracellular production of physiological Reactive Oxygen Species (ROS). The latter are highly reactive compounds due to the presence of shell electrons with unpaired valence. The most relevant radicals are OH and H_2_O_2_. ROS are formed as natural products of normal oxygen metabolism and play important roles in cells signaling and homeostasis. However, during inflammation, ROS levels can dramatically increase. This may result in the increase of oxidizing conditions, thus leading to cell structures damage. Cumulatively, this is known as *oxidative stress*[2]. Normally, cells defend themselves against ROS damage with enzymatic and non-enzymatic systems. Alpha-1-microglobulin, superoxide dismutases, catalases, lactoperoxidases, glutathione peroxidases and peroxiredoxins are considered an important enzymatic system. Moreover, small molecules such as ascorbic acid (vitamin C), tocopherol (vitamin E), uric acid and glutathione also play important roles as non-enzymatic systems [2,3].

Effects of ROS on cell metabolism are well documented. The effects of ROS are not limited to the apoptosis but also include positive actions, such as the induction of host defence genes [4] and the mobilization of ion transport systems [2]. Platelets involved in wound repair and blood homeostasis release ROS to recruit additional platelets in the sites of injury. In addition, they provide a link to the adaptive immune system via the recruitment of leucocytes [5].

ROS are implicated in the cellular activity as well as in variety of inflammatory responses; furthermore, they can potentially cause cellular injury by the damage of DNA, RNA, proteins, lipids, which contributes to ageing and other pathologies like cardiovascular diseases, neurodegeneration and periodontal diseases [6]. The free radical theory of aging assumes that there is a single basic cause of aging, modified by genetic and environmental factors, and postulates that free radical reactions are involved in aging and age-related disorders [2,4,6].

Soory et al. claim that an increase in ROS can induce a hyper-inflammatory status in aggressive forms of periodontal diseases, leading to an unbalance of redox status, that results in a cellular damage [7] .

In addition to ROS, the Advanced Glycation End products (AGEs) are another emerging marker of oxidative stress. The AGEs are heterogeneous products, that are constantly formed in physiological conditions, but significantly increase in hyperglycaemia and excessive oxidative stress [8,9]. Recent studies suppose that the AGEs are involved in a large number of systemic diseases, where the oxidative component is strong, such as in diabetes and hypertension [8].

The activation of these pathways is not restricted to limited areas of the body; in fact, their signaling triggers systemic responses, which are also visible in teeth-supporting tissues. Indeed, it is documented that AGEs induce the activation of pro-inflammatory molecules such as TNFalfa, Interleukin 6, 10 (IL-6, IL-10) and C-Reactive Protein (CRP). These molecules are strongly involved in host response against periodontal pathogens and promote the osteoclast activation in periodontitis [6].

The activation of these complexes leads to the interaction between AGEs and RAGE cellular receptors (found in many cell populations), which amplify the release of cytokines, metalloproteinases (MMPs) and ROS [10]. The pathogenetic action of these compounds, in fact, performs directly, damaging the tissues, or indirectly, through the binding of a specific receptor, called RAGE, which belongs to the family of immunoglobulins [10]. This receptor is physiologically but poorly present in many cells, while it is over-expressed in conditions such as diabetes, vasculopathy and cancer [11]. The AGE-RAGE bond produces a cascade of pro-inflammatory signaling with subsequent activation of redox-sensitive transcription factors, such as NF-kB [12]. This interaction produces a hyper-permeability at the level of endothelial cells and activates the VCAM-1 molecules, whereas on monocytes it contributes to chemotaxis and to the increase of cytokines, such as the Tumor Necrosis Factor (TNF), and interleukins IL-1 and IL-6 [13]. Collagen synthesis by fibroblasts is also reduced [14].

From this basis, in this preliminary case series/control study we investigate the presence of AGEs and oxidative stress in periimplantitis, in order to deepen this poorly understood field.

## Methods

This study was conducted in accordance with the Declaration of Helsinki. The Committee on Ethics in Science of the University of L’Aquila approved the study and written informed consent was obtained from each subject.

Due to the lack of data about this topic, we decided at first to elucidate the possibility that oxidative stress plays a role not only in periodontitis, but also in periimplantitis. From this basis, we needed to compare a group of people with failed dental implant with a first group of people with chronic periodontal disease and a second group of healthy people. So, three groups were examined: *Group 1* (periimplantitis), *Group 2* (chronic periodontal disease) and *Group 3* (healthy subjects), for a total number of 15 patients.

According to literature, the peri-implant tissues were compared with periodontal tissues of healthy and chronic parodontopathic subjects, as suggested by Fritz et al. [15].

The enrolled patients shared the following exclusion criteria: moderate/severe hypertension (according to American Heart Association), metabolic syndrome, alcoholism, history of antibiotics/anti-inflammatories/other medications consumed in the past 6 months, drugs, diabetes, vegetarian diet, autoimmune diseases, liver and kidney diseases, cancer, metastasis, osteoporosis, radiographic evidence of bone loss, hypovitaminosis D, dyslipidemia, smoking, history of systemic diseases.

For each group, moreover, there were specific inclusion criteria:

• *Group 1 (periimplantitis)* - dental implants, placed using traditional submerged technique and failed within 6 months after implantation.

• *Group 2* (chronic periodontal disease) – age-matched patients who needed dental extraction for chronic periodontitis. Mean of probing depth ≥ 5 mm (six conventional sites) with RX evidence of alveolar bone loss.

• *Group 3* (healthy subjects) – age-matched patients who needed dental extraction for dental trauma without history of periodontal diseases.

The same operator who had placed the implants removed them after diagnosis of periimplantitis in order to mantain intraoperator repeatability. As described by literature, different production lots were used in order to reduce the mechanical defects bias of the implants. Diagnosis of periimplantitis was made according to Lindhe et al. using repeated measures over time of probing depth (PD) of conventional six sites and RX [1].

### Implants collection method

Five dental implants, failed within 6 months after implantation (3 from mandible, 2 from maxilla), were taken from 5 subjects (3 M/2 F) aged between 43–57 years (average 49.6 ± 4.6). Peri-implant tissues adherent to the implant spires were conserved; the contamination of the implants by oral cavity was prevented. Samples were stored in Phosphate Saline Buffest (PBS) pH 7,4 (P5368; Sigma–Aldrich, St. Louis, MO, USA) before dry freezing at -80°C.

### Tooth collection method

Both in the periodontopatic and the healthy group, dental extraction followed the same procedure. After local anesthesia (Mepivacaine 2% and adrenaline 1:100.000 – Scandonest 2% Ogna Laboratori Farmaceutici - Milan), teeth were extracted and then put in PBS solution before dry freezing at -80°C. We collected 5 teeth from as many subjects with chronic periodontal disease (2 M/3 F) aged between 45–53 years (average 49.2 ± 2.9) and 5 teeth from healthy subjects (3 M/2 F) aged between 37–51 years (average 45.0 ± 5.8).

### Saliva collection method

Whole saliva of all enrolled patients was also collected to assess oxidative stress analysis. Saliva was collected before any oral operation, in the morning, after a vigorous rinsing with water, using Salimetrics**^®^** collection system (Salimetrics UK - Oral Swab - Swab Storage Tube) [16].

### Tissues sample processing: extraction of periodontal and peri-implant tissues

According to Takatsu et al. [17], the same method for tissues extraction was used in the samples. Both implants and teeth were unfrozen by bain-marie at 37°C for 5 min. First, the samples were processed with scalpel to remove the small quantity of apical and coronal tissues into the Petri dish with PBS 1x. The periodontal ligament and the peri-implant tissues were so obtained, and then dry frozen at -80°C.

### SDS-Page Electrophoresis and Western Blotting of extracted tissues

The tissues pellet were unfrozen using bain-marie at 37°C for 5 min. Three wash cycles with PBS 1x at 4000RPM/10 min were done. The cells lysation was assessed in ice for 20 min using 200 uL of standard RIPA buffer (R0278; Sigma–Aldrich, St. Louis, MO, USA). Every 5 minutes vortexing was done. Three cycles of freezing-unfreezing with liquid nitrogen and three cycles of sonication in ice were assessed (10 sec for 60 sec of recovery). Bradford method was used for the proteins quantification.

Standard SDS-Page and Western Blotting were done using monoclonal antibody for AGEs (AGE06B – Biologo.de). Silver stained was performed on Polyacrylamide gel.

### Saliva analysis

According to producer (Salimetric**^®^** UK), the vials containing saliva were unfrozen and centrifuged at 6000RPM/10 min to obtain whole saliva for analysis.

Oxidative stress was measured as lipid peroxidation using specific colorimetric assay for ThioBarbituric Acid Reactive Substances - TBARS – (BioAssay USA, DTBA-10). The TBARS absorbance, for each sample, was measured three times and the mean value was taken as oxidative stress level, as suggested by the producer.

## Results

The statistical analysis for oxidative stress was performed using software SPSS (Version 20.0 for Windows 7). Statistical difference in oxidative stress in terms of TBARS light absorbance between groups was analyzed by one-way ANOVA with post-hoc Bonferroni correction due to the small sample size, even though the Shapiro-Wilk test revealed a normal distribution of data. The level of significance was set at p < 0.05 for all tests. Results are expressed as mean and standard deviation (Table 1).

**Table 1:** Results of salivary TBARS assey

**Groups**	**Abs**
*Health*	*0,62 (0,22)*^a^
*Peri Implantitis*	*1,25 (0,11)*^b^
*Chronic Periodontitis*	*1,70 (0,12)*^c^

Mean of absorbance (Abs) and standard deviation (SD) in groups. Different superscript letters indicates statistical significant difference (P < 0.05) between groups performing one way ANOVA with post-hoc Bonferroni.

**Figure 1:**
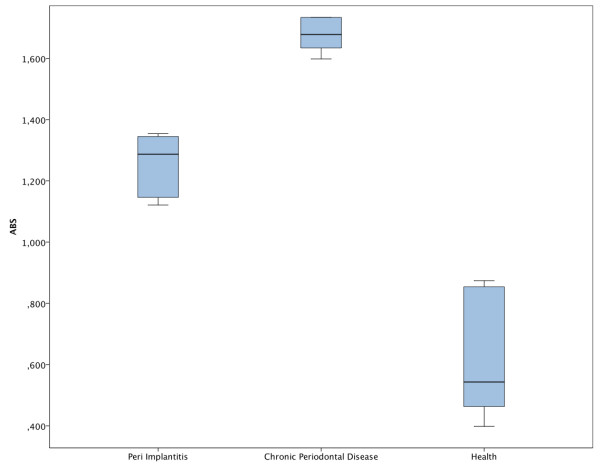
Box blot of salivary TBARS in the study groups.

The results showed statistically significant differences in each group. In particular, the chronic periodontal disease group showed higher oxidative stress than periimplantitis and healthy groups. Periimplantitis group compared to the healthy one had higher oxidative stress levels (p < 0,001). Chronic periodontal disease compared to periimplantitis showed higher oxidative stress (p = 0,002) as described in Figure 1. Chronic periodontal disease compared to health met the expectation, showing even higher (p < 0,0001) oxidative stress levels than compared to periimplantitis.

**Figure 2:**
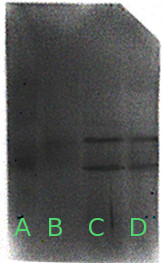
**Western Blotting image of analyzed tissues. A) **Molecular weight; **B)** healthy subjects; **C) **periimplantitis; **D) **chronic periodontal disease.

Western Blotting analysis showed that AGEs are present in both tissues of periimplantitis and periodontopatic groups compared to healthy subject tissues (Figure 2).

## Discussion

With the limitation of the sample size, in this preliminary study we highlighted the possible role of AGEs and oxidative stress in the genesis and progression of periimplantitis. From our data a clear picture emerges, in which oxidative stress and consequent AGEs production are possibly involved in the failure of dental implants. This hypothesis is strongly supported by recent literature, since a possible role of AGEs and oxidative stress was established in many oral inflammatory diseases [6].

One of our recent reviews of literature also supports the hypothesis that glycation and oxidative stress are the possible common link with periodontal diseases in patients with metabolic syndrome, which is an example of systemic proinflammatory condition [8]. Minor pro oxidant conditions, too, like moderate smoking, unbalanced diet, mild hypertension, minor or undiagnosed hyperglycemia, can promote ROS production and irreversible accumulation in genetically predisposed patients [8]. Finally, genetic conditions probably play an important role in the failure of dental implants and would explain why it can manifest in some patients without apparent risk factors for periimplantitis. Many studies, in fact, support the hypothesis that the accumulation of ROS is influenced by genetic factors. Motohashi et al., for example, showed that the mutation of the gene Nrf2 promotes ROS accumulation [18].

The ROS production induces formation of AGEs, which irreversibly accumulate in the peri-implant tissues, and this may induce tissue damage by both affecting the functional status of collagen fibers and promoting an increase of oxidative stress and inflammation. These effects are mediated by AGE-RAGE interaction. From its side, AGEs formation in the extracellular matrix may contribute to increase the production and release of ROS from peri-impant tissues cells and phagocytes, with subsequent induction of proinflammatory cytokines and metalloproteinases (MMPs), leading to osteoblast inactivation and consequent osteoclast activation [6].

Our findings are in line with literature, since we found higher values of oxidation in periodontitis than in healthy people, and also add new data about the unexplored field of oxidation/glycation in periimplantitis. Our preliminary data, in fact, showed that periimplantitis is characterized by an increase in oxidative stress markers in saliva and higher levels of AGEs in periimplant tissues. These alterations are similar in severity to those of periodontitis, even though they don’t reach the same values. The quantity of oxidation in periimplantitis group, in fact, is less than in periodontitis group. This finding is coherent with the fact that periodontal disease is a chronic disorder, with an early beginning, a major duration and, probably, a systemic involvement, of which they may be the mirror.

Also the Western Blotting analysis of AGEs showed more glycation in periimplantitis than in healthy people. This finding supports and strengthens the previous data, confirming the involvement of the oxidizing system and the AGE-RAGE interaction in periimplantitis.

Based on the data reported, a potential innovative therapy of periimplantitis may be hypothesized, using drugs with antioxidant and anti-AGEs effects. Possible interventions against AGEs formation and AGE-RAGE mediated damage are numerous. The discovery of chemical agents that can inhibit glycation reactions may have potential therapeutic importance. Pyridoxamine, one of the neutral form of vitamin B, has been shown to inhibit AGEs formation by interfering with post Amadori oxidative reactions, and it is employed in clinical trial evaluating the efficacy of pyridoxamine in inhibiting the progression of proteinuria and hyperlipidemia in diabetic patients [19]. A further inhibitor of protein glycation is metformine, that has additional effects on AGEs accumulation by reducing oxidative stress [20]. Recently it has been reported that nifedipine and telmisartan, respectively a Ca^2+^ channel blocker and an angiotensin receptor antagonist, exert anti-oxidative and anti-AGE-RAGE axis properties [21].

A further group of anti-AGE drugs may be that of modified tetracyclines with antioxidant effects. In addition to their antimicrobial action, these molecules are effective in combating oxidative stress in periodontal disease and have beneficial effects on systemic diseases driven by oxidative stress [22]. This is the case of doxycycline, whose efficacy in reducing periodontal inflammation has been associated with a reduction in level of glycated haemoglobin in diabetics [23].

In addition to drugs therapy, also diet and life style can provide to reduce oxidative stress and AGEs formation. In fact, flavonoids, present in many foods like green tea, chocolate and fruit, have the capacity to reduce glycation [23]. Also vitamins C, E, P have antioxidant proprieties [24]. Therefore, it is clear how dietary factors may play an important role in the strategy against the oxidation.

## Conclusion

This preliminary study, with the limitation of the sample size, identifies the periimplantitis as a multifactorial disease, in which glycation and oxidative stress play a role in terms of etiology and severity. This finding remarks the recent literature assumptions, that support a closer correlation between systemic conditions and oral health. The molecular pathways that are involved in periimplantitis are unknown, but probably oxidizing is involved in its etiology, as it is in the periodontitis.

These data suggest to the clinician a new strategy to prevent the failure of dental implants. Probably, in a not too distant future, the simple measurement of oxidative stress or AGEs in saliva, prior to dental implant surgery, could prevent implant failure. To this aim, future investigation on the presence of AGEs and oxidizing in the periimplantitis is needed, in order to develop prevention and treatment strategies for this disease. However, common and safe drugs or target diets could be used in order to prevent or treat the periimplantitis.

## Competing interests

All authors declare that they have no competing interests.

## Authors’ contributions

DP an AM contributed to the conception and design of the study, the analysis and interpretation of the data and drafted the manuscript. EO, MS and IC were involved in the interpretation of the data and contributed to the revision of the drafted manuscript. RG provided statistical analysis. All authors read and approved the final manuscript.
